# Ergothioneine Prevents Neuronal Cell Death Caused by the Neurotoxin 6-Hydroxydopamine

**DOI:** 10.3390/cells13030230

**Published:** 2024-01-25

**Authors:** Saho Yuzawa, Motonari Nakashio, Suzuna Ichimura, Mikako Shimoda, Ayaka Nakashima, Yuka Marukawa-Hashimoto, Yusuke Kawano, Kengo Suzuki, Kenichi Yoshitomi, Masahiro Kawahara, Ken-ichiro Tanaka

**Affiliations:** 1 Laboratory of Bio-Analytical Chemistry, Research Institute of Pharmaceutical Sciences, Faculty of Pharmacy, Musashino University, 1-1-20 Shinmachi, Nishitokyo, Tokyo 202-8585, Japan; s1943100@stu.musashino-u.ac.jp (S.Y.); s1743112@stu.musashino-u.ac.jp (M.N.); s1943008@stu.musashino-u.ac.jp (S.I.); s1343142@stu.musashino-u.ac.jp (M.S.); makawa@musashino-u.ac.jp (M.K.); 2 Euglena, Co., Ltd., 5-29-11 G-BASE Tamachi 2nd Floor Shiba, Minato-ku, Tokyo 108-0014, Japan; nakashima@euglena.jp (A.N.); hashimoto@euglena.jp (Y.M.-H.); suzuki@euglena.jp (K.S.); 3 Sakichi, Co., Ltd., 5-531 Kuromaru-Machi, Omura, Nagasaki 856-0808, Japan; sakichi@sakichi-inc.jp

**Keywords:** ergothioneine, neuronal cell death, 6-OHDA, oxidative stress, functional food

## Abstract

Neuronal cell death is a key mechanism involved in the development and exacerbation of Parkinson’s disease (PD). The excessive production of reactive oxygen species (ROS) is a major cause leading to neuronal death; therefore, compounds that prevent oxidative stress-dependent neuronal death may be promising as a preventive method for PD. Ergothioneine is a natural amino acid with antioxidant properties, and its protective functions in the body are attracting attention. However, there has been no investigation into the protective functions of ergothioneine using in vivo and in vitro PD models. Thus, in this study, we analyzed the efficacy of ergothioneine against 6-hydroxydopamine (6-OHDA)-dependent neuronal cell death using immortalized hypothalamic neurons (GT1-7 cells). First, we found that ergothioneine prevents 6-OHDA-dependent neuronal cell death by suppressing ROS overproduction in GT1-7 cells. The cytoprotective effect of ergothioneine was partially abolished by verapamil, an inhibitor of OCTN1, which is involved in ergothioneine uptake. Furthermore, ergothioneine-rich Rice-koji (Ergo-koji) showed cytoprotective and antioxidant effects similar to those of ergothioneine. Taken together, these results suggest that ergothioneine or foods containing ergothioneine may be an effective method for preventing the development and progression of PD.

## 1. Introduction

Parkinson’s disease (PD) is one of the most common progressive neurodegenerative diseases. PD affects the central nervous system and the parts of the body innervated by affected nerves, inducing movement disorders such as resting tremor, akinesia, impaired postural reflexes, and muscle rigidity. Difficulty walking and slow movements are typical symptoms seen in the early stages of PD, and patients gradually lose the ability to walk on their own and are forced to live in a wheelchair or are bedridden as PD progresses [1,2]. To compensate for dopamine depletion (due to the decrease in dopaminergic neurons), the dopamine precursor L-dopa, anticholinergic drugs, and amantadine hydrochloride are generally used for treating patients with PD. These drugs have shown some efficacy [3], but there is no established method to prevent the development and progression of PD.

Although the pathogenesis of PD is not fully understood, the loss of dopaminergic neurons in the substantia nigra of the midbrain and an exaggerated inflammatory response by microglia in the lesion area are thought to be major causes of PD [4,5]. Treatment of mice or neurons with 6-hydroxydopamine (6-OHDA) or N-methyl-4-phenylpyridinium iodide (MPP+) have been used in basic research because they can produce models that mimic oxidative stress-mediated dopaminergic neuronal degeneration and increased microglial inflammation [6,7]. Moreover, dysfunction of the hypothalamic–pituitary–adrenal axis, which controls the secretion of various hormones, is reportedly involved in the development and progression of PD [8]. Plasma concentrations of nocturnal growth hormone and adrenocorticotropic hormone are decreased in patients with PD compared with healthy controls, which indicates there are hypothalamic disturbances in PD [9]. One study found a reduced number of neurons in the hypothalamus of patients with PD [10]. There is also a significant positive correlation between a decrease in neurons containing orexin or melanin-concentrating hormone in the hypothalamus and the clinical stage of PD [11]. These previous findings suggest that inhibiting neuronal death in the hypothalamus may be important in preventing the development and progression of PD.

Ergothioneine is a rare, naturally occurring amino acid that is produced by actinomycetes, cyanobacteria, and certain fungi and is abundant in mushrooms such as tamogi and shiitake mushrooms. Ergothioneine is resistant to heat and pH changes, and because it has a thione group with a sulfur molecule doubly bonded to a carbon (thiourea derivative), it reacts with reactive oxygen species (ROS), such as hydroxyl radicals and has high antioxidant activity [12,13]. Ergothioneine is present throughout the human body, including in the liver, kidneys, brain, and red blood cells [14], but it cannot be synthesized in the human body, so it must be obtained from food or supplements. Recently, an organic cation transporter, novel type 1 (OCTN1), a carnitine/organic cation transporter, was discovered as a membrane protein that transports ergothioneine into the cell [15,16]. Consequently, the biological functions of ergothioneine in the body have attracted increasing attention. For example, Yang et al. analyzed the efficacy of orally administered ergothioneine against amyloid β (Aβ)1-40-dependent neuronal injury in the hippocampus of mice. They found that ergothioneine significantly reduced escape latency and increased the frequency of successful avoidance in both long-term memory avoidance and water maze tests. It also significantly inhibited Aβ accumulation and lipid peroxidation in the hippocampus [17]. Furthermore, another group analyzed the efficacy of intracerebroventricular or intraperitoneal administration of ergothioneine in a rat stroke model. They found that ergothioneine significantly reduced cerebral infarct volume with both methods of administration, while MRI analysis revealed that ergothioneine had neuroprotective effects in the stroke model [18].

Although several biological functions of ergothioneine have been described, its efficacy against 6-OHDA-dependent hypothalamic neuronal cell death has not been investigated. Therefore, in this study, we examined the efficacy of ergothioneine on 6-OHDA-dependent neuronal cell death using immortalized hypothalamic neurons and GT1-7 cells. We also determined the effect of ergothioneine on 6-OHDA-dependent ROS production and the efficacy of ergothioneine-rich Rice-koji (Ergo-koji) on 6-OHDA-dependent neuronal cell death.

## 2. Materials and Methods

### 2.1. Chemicals and Reagents

Ergothioneine was kindly provided by Tetrahedron (Paris, France). 6-OHDA was purchased from R&D Systems, Inc. (Minneapolis, MN, USA). Ergo-koji was provided by Euglena, Co., Ltd. (Tokyo, Japan). The ergothioneine content in Ergo-koji is 73.6 mg/100 g. Ergo-koji is fermented with euglena powder (Euglena Co., Ltd., Tokyo, Japan) in the fermentation stage of Rice-koji (Sakichi Co., Ltd., Nagasaki, Japan). The addition of euglena powder ensures a higher ergothioneine content of Ergo-koji than fermentation without euglena powder. MPP+ was purchased from Cayman Chemical (Ann Arbor, MI, USA). CellTiter-Glo^®^ 2.0 was purchased from Promega Corporation (Madison, WI, USA). Dulbecco’s Modified Eagle’s Medium/Ham’s Nutrient Mixture F-12 (DMEM/Ham’s-F12) was purchased from Fujifilm Wako Pure Chemical Corporation (Tokyo, Japan). 2′,7′-Dichlorodihydrofluorescein diacetate (H_2_DCFDA) was obtained from Merck KGaA (Darmstadt, Germany). The FastGene™ RNA Basic kit was obtained from Nippon Genetics Co., Ltd. (Tokyo, Japan), PrimeScript^™^ RT master mix (Perfect Real Time) was obtained from Takara Bio (Shiga, Japan), and THUNDERBIRD^®^ Next SYBR^®^ qPCR mix was obtained from Toyobo (Osaka, Japan).

### 2.2. Cell Culture

GT1-7 cells are immortalized hypothalamic neurons derived from mice and were provided by Dr. R. Weiner (University of California, San Francisco, CA, USA). GT1-7 cells were cultured in DMEM/Ham’s-F12 medium supplemented with 10% fetal bovine serum (South American origin) (Biowest, Nuaillé, France). After treatment with trypsin (Fujifilm Wako Pure Chemicals), the cells were suspended in a serum-free medium, seeded into culture plates, and cultured in a humidified incubator (7% CO_2_) at 37 °C [19].

### 2.3. Determination of Cell Viability

Cell viability was measured as in our previous studies [20,21]. Briefly, GT1-7 cells were seeded onto 96-well culture plates at a concentration of 3.0 × 10^4^ cells/well in a 200 μL culture medium. After 24 h of preincubation, 6-OHDA (final concentrations from 0 µmol/L to 80 µmol/L) or MPP+ (final concentrations from 0 mmol/L to 8 mmol/L) was added to the medium. Alternatively, after 24 h of preincubation, the cells were treated with ergothioneine (final concentrations from 0 mmol/L to 1.0 mmol/L) 10 min before 6-OHDA (final concentration: 40 mmol/L) or MPP+ (final concentration: 4 mmol/L) was added to the medium. After incubation for 24 h, cell viability was measured using CellTiter-Glo^®^ 2.0, a luminescent reagent that measures intracellular ATP. Assuming that ergothioneine is taken as a daily meal or supplement, ergothioneine was pretreated to GT1-7 cells, and its efficacy was analyzed in this study. Furthermore, although there is variation among reports [22], the blood concentration of ergothioneine in humans is about 0.2 mmol/L, which is comparable to the concentration of ergothioneine used in this experiment.

### 2.4. Measurement of ROS Levels

GT1-7 cells were pre-cultured in black 96-well microplates (3.0 × 10^4^ cells/well) for 24 h. Thereafter, the cells were incubated with the ROS indicator, H_2_DCFDA (10 µmol/L), for 60 min. The cells were then treated with ergothioneine (final concentrations from 0 mmol/L to 1.0 mmol/L) prior to the addition of 6-OHDA (final concentration: 40 μmol/L) to the medium. After 1 h, ROS levels were measured using a microplate reader (Tecan, Kawasaki, Japan; excitation: 480 nm, emission: 530 nm).

### 2.5. Real-Time Reverse Transcription Polymerase Chain Reaction (RT-PCR) Analysis

Total RNA was extracted from GT1-7 cells using the FastGene™ RNA Basic kit in accordance with the manufacturer’s protocol. Samples were reverse transcribed using the PrimeScript RT master mix. The resulting cDNA was used in real-time PCR experiments with THUNDERBIRD Next SYBR qPCR mix and analyzed on a Bio-Rad CFX96™ real-time system (Hercules, CA, USA) with CFX Manager™ software (Version 3.1). Specificity was confirmed by electrophoretic analysis of reaction products, with the inclusion of template- or reverse transcriptase-free controls. To normalize the amount of total RNA present in each reaction, glyceraldehyde-3-phosphate dehydrogenase (*Gapdh*) cDNA was used as an internal standard. Primers were designed using the Primer-BLAST website (https://www.ncbi.nlm.nih.gov/tools/primer-blast/ (accessed on 16 November 2023)). Primer sequences are shown in Supplementary Figure S1.

### 2.6. Statistical Analysis

All data are expressed as mean ± standard error of the mean (S.E.M.). One-way analysis of variance (ANOVA) followed by Dunnett’s test or Student’s *t*-test for unpaired results was used to examine differences between three or more groups or between two groups, respectively. Mac statistical analysis Ver. 3.0 software (Esumi Co., Ltd., Tokyo, Japan) was used for all statistical analyses. Differences were considered to be significant when *p* < 0.05 (* or # *p* < 0.05, ** or ## *p* < 0.01). Details of the symbols are given in the figure legends. The number of samples (n) is also indicated in each figure.

## 3. Results

### 3.1. Efficacy of Ergothioneine on 6-OHDA-Induced Neuronal Cell Death

**Figure 1:**
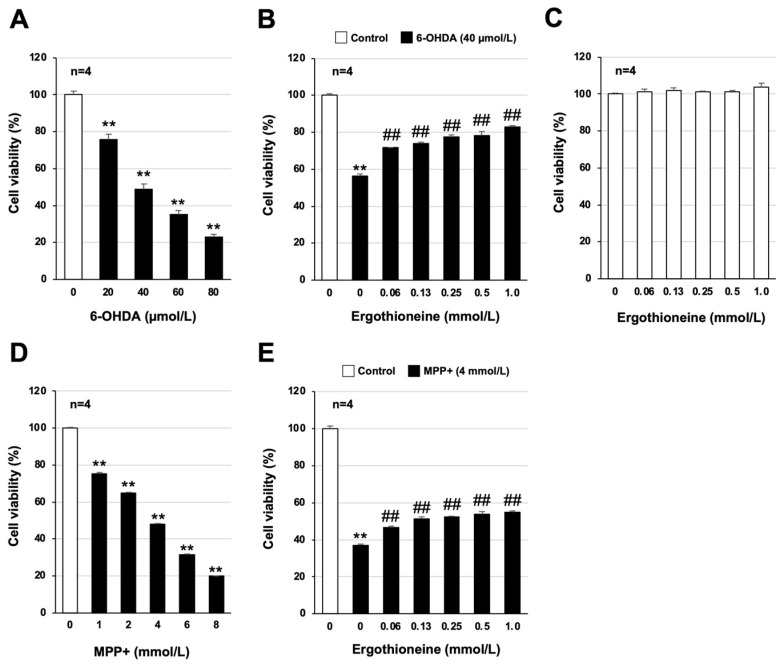
Neuroprotective effect of ergothioneine. GT1-7 cells were treated with the indicated concentrations of 6-hydroxydopamine (6-OHDA) (μmol/L) or N-methyl-4-phenylpyridinium iodide (MPP+) (mmol/L) and incubated for 24 h (**A**,**D**). GT1-7 cells were pretreated with ergothioneine (0.06–1.0 mmol/L) and then incubated in the absence (Control) or presence of 6-OHDA (40 µmol/L) or MPP+ (4 mmol/L) for 24 h (**B**,**E**). GT1-7 cells were treated with ergothioneine (0.06–1.0 mmol/L) alone and cultured for a further 24 h (**C**). Cell viability was measured using CellTiter-Glo^®^ 2.0. Values represent mean ± S.E.M. (*n* = 4). ** *p* < 0.01, vs. Control; ## *p* < 0.01, vs. 6-OHDA (40 µmol/L) alone or MPP+ (4 mmol/L) alone.

Neuronal cell death is a key mechanism in the development and exacerbation of PD [4,5]. In addition to inducing various PD-like symptoms when 6-OHDA is administered to the brains of experimental animals, 6-OHDA acts as a neurotoxin and induces neuronal cell death in cellular experimental systems [6,23]. First, we measured the viability of GT1-7 cells (an immortalized strain of mouse hypothalamic neurons) treated with 6-OHDA alone. Treatment of GT1-7 cells with final concentrations of 20, 40, 60, and 80 μmol/L of 6-OHDA resulted in a concentration-dependent decrease in cell viability (Figure 1A). Cell viability after treatment was 75.9 ± 2.7, 48.8 ± 2.7, 35.2 ± 2.3, and 22.9 ± 1.6% (mean ± S.E.M., *n* = 4), respectively, and all groups showed a significant decrease in cell viability compared with the control group treated with ultrapure water. We next analyzed the effect of ergothioneine pretreatment on this 6-OHDA-induced reduction in cell viability; the final concentration of 6-OHDA was 40 μmol/L, which reduces cell viability by approximately 50%. Pretreatment with ergothioneine significantly ameliorated the 6-OHDA-dependent decrease in GT1-7 cell viability (Figure 1B). The effect was dependent on the concentration of ergothioneine, with cell viability restored to 78.1 ± 2.5 and 82.8 ± 0.8% (mean ± S.E.M., *n* = 4) when pretreated with 0.5 mmol/L and 1.0 mmol/L of ergothioneine, respectively. Under these experimental conditions, treatment with ergothioneine alone had little effect on cell viability (Figure 1C). Similar to 6-OHDA, MPP+ acts as a neurotoxin in both in vivo and in vitro experimental systems [7]. Thus, we next determined whether ergothioneine also has an inhibitory effect on MPP+-dependent neuronal cell death. Treatment of GT1-7 cells with 1.0–8.0 mmol/L MPP+ resulted in reduced cell viability (Figure 1D). Furthermore, the effect of ergothioneine on 4.0 mmol/L MPP+-dependent neuronal cell death was analyzed, in which cell viability was 36.9 ± 0.7% (mean ± S.E.M., *n* = 4) (Figure 1E). Ergothioneine significantly ameliorated this MPP+-dependent reduction in cell viability, with cell viability of 54.0 ± 1.3 and 54.9 ± 0.5% (mean ± S.E.M., *n* = 4) when pretreated with 0.5 mmol/L and 1.0 mmol/L ergothioneine, respectively (Figure 1E).

**Figure 2:**
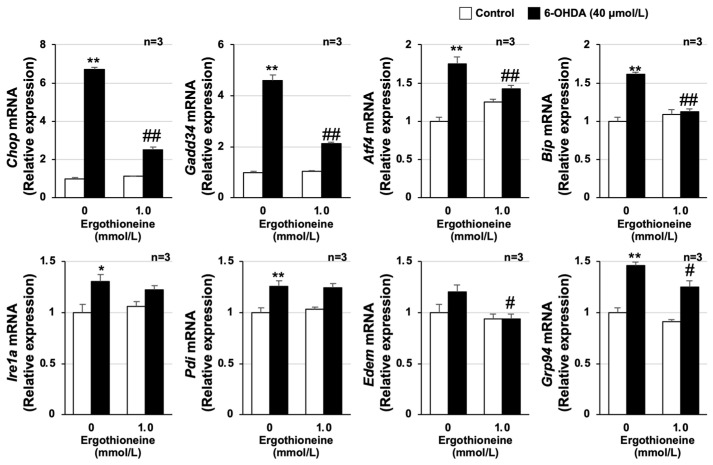
Ergothioneine suppresses the 6-hydroxydopamine-dependent endoplasmic reticulum stress response. GT1-7 cells were pretreated with ergothioneine (1.0 mmol/L) and then incubated in the absence (Control) or presence of 6-OHDA (40 µmol/L) for 24 h. After total RNA extraction from GT1-7 cells, cDNA was synthesized, and real-time RT-PCR was performed using primer pairs that specifically amplify *Chop*, *Gadd34*, *Atf4*, *Bip*, *Ire1α*, *Pdi*, *Edem*, and *Grp94*. Values were normalized to *Gapdh* and expressed relative to control. Values represent mean ± S.E.M. (*n* = 3). * *p* < 0.05, vs. Control; ** *p* < 0.01, vs. Control; # *p* < 0.05, vs. 6-OHDA (40 µmol/L) alone; ## *p* < 0.01, vs. 6-OHDA (40 µmol/L) alone.

Several studies have shown that the endoplasmic reticulum (ER) stress response is a key mechanism of 6-OHDA-dependent neuronal cell death [24,25]. Moreover, our group recently found that the antioxidant peptide, carnosine, and an antioxidant protein–thioredoxin–albumin fusion suppressed 6-OHDA-dependent elevated ER stress-related gene expression in GT1-7 cells [26,27]. Here, we found that 40 µmol/L of 6-OHDA treatment increased the expression of ISR-related genes, in particular, the significant upregulation of CCAAT-enhancer-binding protein homologous protein (*Chop*) and growth-arrest and DNA-damage-inducible gene 34 (*Gadd34*) expression. Specifically, *Chop* and *Gadd34* mRNA increased by 6.72 ± 0.08-fold and 4.60 ± 0.22-fold (mean ± S.E.M., *n* = 3), respectively, compared with the control group. In addition, apart from ER degradation enhancing α mannosidase (*Edem*), activating transcription factor 4 (*Atf4*), binding immunoglobulin protein (*Bip*), inositol-requiring transmembrane kinase/endoribonuclease 1α (*Ire1α*), protein disulfide isomerase (*Pdi*), and glucose-regulated protein 94 (*Grp94*) were all significantly increased by 6-OHDA treatment. In contrast, ergothioneine pretreatment suppressed the upregulation of these genes, especially *Chop* (to 2.52 ± 0.15-fold) and *Gadd34* (to 2.13 ± 0.04-fold) mRNA (mean ± S.E.M., *n* = 3). These results suggest that ergothioneine prevents 6-OHDA-induced neuronal cell death by suppressing the increased expression of ER stress-related genes (Figure 2).

### 3.2. Efficacy of Ergothioneine on 6-OHDA-Induced Reactive Oxygen Species (ROS) Production

**Figure 3:**
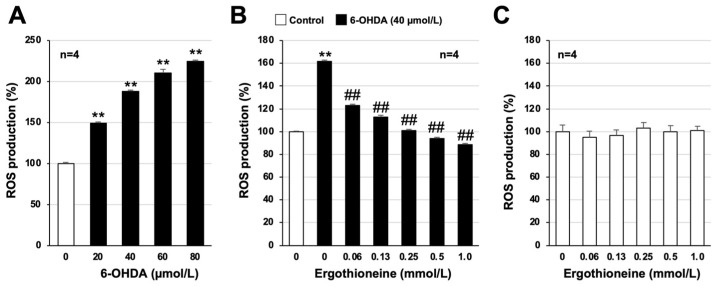
Antioxidant effect of ergothioneine in GT1-7 cells. GT1-7 cells were pretreated with a reactive oxygen species (ROS) indicator, 2′,7′-dichlorodihydrofluorescein diacetate (H_2_DCFDA) (10 µmol/L) for 60 min (**A**,**B**). Cells were then treated with 6-OHDA (20–80 μmol/L) and cultured for 1 h (**A**). GT1-7 cells were pretreated with ergothioneine (0.06–1.0 mmol/L) and then incubated in the absence (Control) or presence of 6-OHDA (40 µmol/L) for 1 h (**B**). GT1-7 cells were treated with ergothioneine (0.06–1.0 mmol/L) alone and cultured for 1 h (**C**). ROS levels were measured using a fluorescence microplate reader. Values represent mean ± S.E.M. (*n* = 4). ** *p* < 0.01, vs. Control; ## *p* < 0.01, vs. 6-OHDA (40 µmol/L) alone.

It was suggested that the production of ROS may contribute to the induction of neuronal cell death and inflammatory responses in PD, ultimately invoking the onset and progression of PD [28,29]. Furthermore, in our recent study, we reported that 6-OHDA treatment induced ROS production in GT1-7 cells [26,27]. Thus, we analyzed the effect of ergothioneine on 6-OHDA-induced ROS production using an intracellular ROS detection reagent. Treatment with 6-OHDA alone significantly increased ROS levels in GT1-7 cells in a concentration-dependent manner (Figure 3A), similar to the results of previous studies [27]. Moreover, 40 µmol/L of 6 OHDA treatment increased ROS production to 188.0 ± 1.9% (mean ± S.E.M., *n* = 4) when the control group value was set to 100%. In contrast, ergothioneine pretreatment significantly inhibited 6-OHDA-dependent intracellular ROS production in a concentration-dependent manner. Under these experimental conditions, ergothioneine treatment alone had little effect on intracellular ROS production (Figure 3C). These results suggest that ergothioneine inhibits 6-OHDA-induced neuronal cell death by suppressing the increase in intracellular ROS levels.

### 3.3. Involvement of OCTN1 in Cytoprotective Effects of Ergothioneine

**Figure 4:**
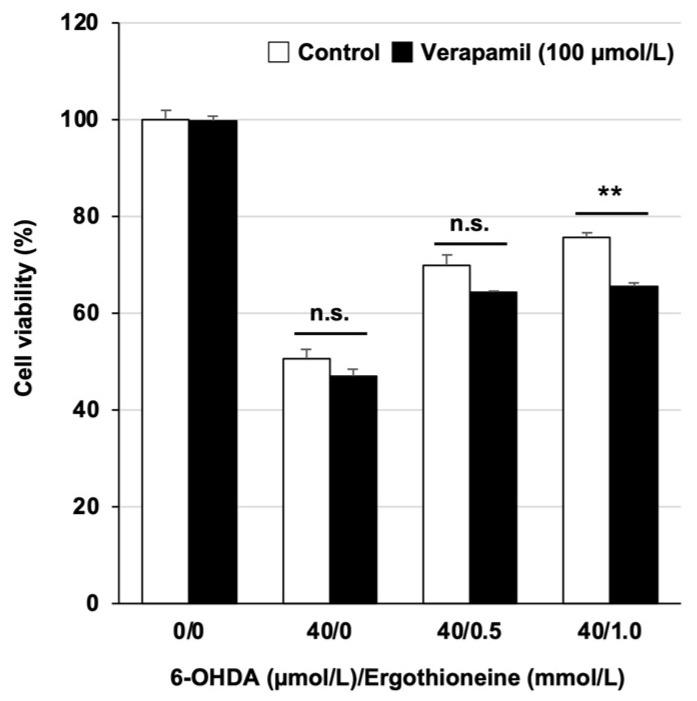
Involvement of OCTN1 in the cytoprotective effect of ergothioneine. GT1-7 cells were pretreated with verapamil (100 µmol/L) for 60 min. After replacement with fresh medium, cells were then pretreated with ergothioneine (0.5 or 1.0 mmol/L) and incubated in the absence (Control) or presence of 6-OHDA (40 µmol/L) for 24 h. Cell viability was measured using CellTiter-Glo^®^ 2.0. Values represent mean ± S.E.M. (*n* = 4). Not Significant (n.s.), ** *p* < 0.01, Control vs. verapamil (100 µmol/L).

The membrane transporter, OCTN1, is a protein that recognizes exogenous or endogenous compounds and transports them in or out of the cell. Several studies in cultured cells and mice have reported that OCTN1 also plays an important role in ergothioneine uptake [15,16]. Therefore, we examined whether the cytoprotective effect of ergothioneine is abolished by verapamil, a known inhibitor of OCTN1 [30]. Similar to previous results, ergothioneine significantly ameliorated the 6-OHDA-induced decrease in cell viability (Figure 4). In contrast, pretreatment with verapamil significantly abolished the protective effect of ergothioneine. Under this experimental condition, treatment with verapamil alone did not affect cell viability and ROS production (Supplementary Figure S2). These results suggest that ergothioneine is transported into the cell via OCTN1 and can exert a cytoprotective effect in GT1-7 cells.

### 3.4. Examination Using Ergothioneine-Rich Rice-Koji (Ergo-Koji)

**Figure 5:**
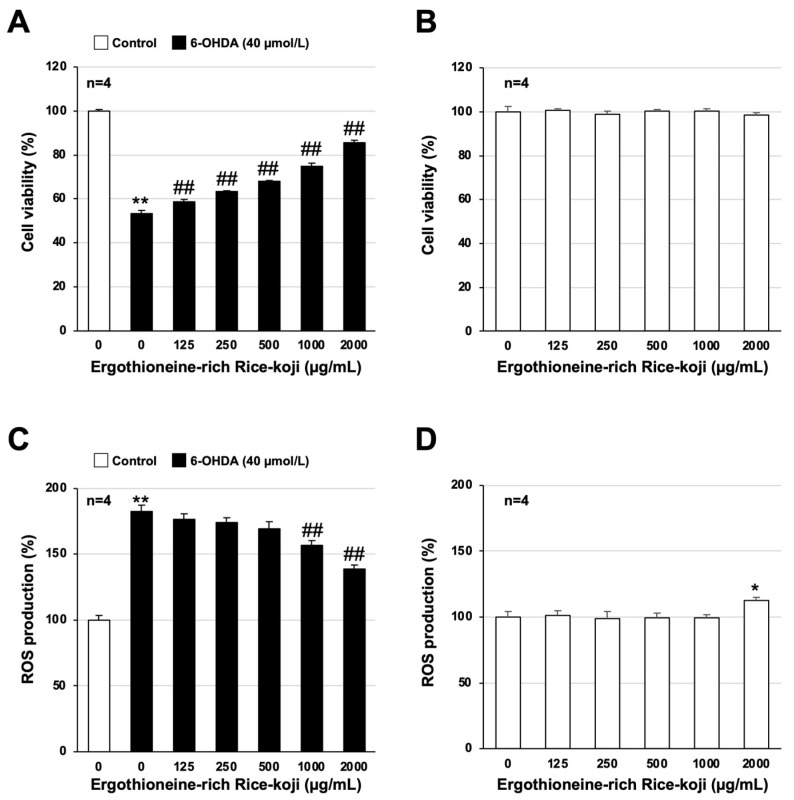
Neuroprotective and antioxidant effects of ergothioneine-rich Rice-koji (Ergo-koji). GT1-7 cells were pretreated with ergothioneine-rich Rice-koji (125–2000 µg/mL) and then incubated in the absence (Control) or presence of 6-OHDA (40 µmol/L) for 24 h (**A**) or 1 h (**C**). GT1-7 cells were treated with ergothioneine-rich Rice-koji (125–2000 µg/mL) alone for 24 h (**B**) or 1 h (**D**). Cell viability was measured using CellTiter-Glo^®^ 2.0 (**A**,**B**). ROS levels were measured using a fluorescence microplate reader (**C**,**D**). Values represent mean ± S.E.M. (*n* = 4). * *p* < 0.05, vs. Control; ** *p* < 0.01, vs. Control; ## *p* < 0.01, vs. 6-OHDA (40 µmol/L) alone.

Several bioactive compounds found in Rice-koji can regulate bodily functions. Ergothioneine is one of the bioactive compounds contained in Rice-koji [31,32]. Therefore, we prepared Ergo-koji (using the method described in the Materials and Methods section) and determined its efficacy against 6-OHDA-dependent neuronal cell death and ROS production. 6-OHDA-dependent cell viability decreased to 53.1 ± 1.7%, while pretreatment with Ergo-koji increased cell viability in a concentration-dependent manner (Figure 5A). In particular, cell viability increased to 74.7 ± 1.6% in the 1000 µg/mL Ergo-koji-treated group and 85.8 ± 1.0% in the 2000 µg/mL Ergo-koji-treated group. 6-OHDA treatment increased intracellular ROS levels to 182.8 ± 4.4%, while pretreatment with Ergo-koji decreased intracellular ROS levels in a concentration-dependent manner (Figure 5C). Furthermore, treatment with Ergo-koji alone had little effect on cell viability and intracellular ROS production (Figure 5B,D). Based on these results, we predict that the ingestion of foods containing ergothioneine will suppress 6-OHDA-induced neuronal cell death and ROS production in the same manner as ergothioneine.

## 4. Discussion

In this study, we used GT1-7 cells as immortalized hypothalamic neurons to determine the protective effect of ergothioneine against 6-OHDA-induced neuronal cell death. We demonstrated that ergothioneine suppresses 6-OHDA-induced cell death and elevated the expression of ER stress-related factors (especially *Chop* and *Gadd34*) in GT1-7 cells. The ER stress response has an important role in the pathogenesis and exacerbation of several diseases, including cancer, pneumonia, and neurological diseases; three ER stress sensors are known: protein kinase R-like ER kinase (PERK), inositol-requiring enzyme-1 (IRE1), and ATF6, through which the unfolded protein response is induced [33,34]. The activation of these sensors triggers various signaling events in the cell. For example, PERK phosphorylates the α subunit of eukaryotic translation initiation factor 2 (eIF2), and the phosphorylated eIF2a affects ATF4 translation. In addition, ATF4 activates transcription of CHOP and GADD34, which are involved in cell death induction [33,34]. Thus, we believe that ergothioneine inhibits 6-OHDA-dependent neuronal cell death by suppressing the expression of ER stress-related factors involved in cell death induction, particularly *Chop* and *Gadd34* and their upstream transcription factor, *Atf4*. In summary, our finding that ergothioneine inhibits 6-OHDA-dependent neuronal cell death via suppression of the ER stress response is novel and highlights the potential of ergothioneine as a preventive method for PD. As 6-OHDA is known to induce apoptosis in neuronal cells [26,29], we would like to analyze the efficacy of ergothioneine by focusing on a more detailed mechanism of cell death induction in the future. Furthermore, although our study shows that the cytoprotective effect of ergothioneine is inhibited by verapamil, an OCTN1 inhibitor, pretreatment with verapamil did not completely eliminate this cytoprotective effect. Moreover, treatment with verapamil alone was cytotoxic at concentrations > 100 µmol/L (Supplementary Figure S2), making it difficult to use in experiments. Therefore, future studies should determine what mechanisms, other than OCTN1, are involved in the cytoprotective effect of ergothioneine.

Oxidative stress (including increased ROS production) is a key factor for inducing neuronal cell death and excessive inflammatory responses in PD [28,29,35]. Indeed, the association between ROS production and PD has been previously shown in clinical studies. One study measured endogenous plasma lipid peroxidation, a marker of oxidative stress, in 52 patients with PD and 40 controls, showing that lipid peroxide levels were 33% higher in the PD group compared with the control group [36]. Akam et al. analyzed 8-hydroxyguanine (8-OHG), a marker of oxidative DNA damage, in the brains of control individuals and patients with PD, finding that 8-OHG levels tended to be elevated in patients with PD, especially in the substantia nigra [37]. In addition, Yoritaka et al. showed in a randomized, double-blind, placebo-controlled, parallel-group pilot study that the administration of coenzyme Q10, which has antioxidant properties, reduced scores associated with the severity and progression of PD compared with a placebo group [38]. Moreover, Monti et al. evaluated the clinical effects of N-acetylcysteine (NAC), a precursor of the antioxidant glutathione, in patients with PD. The results showed that NAC administered intravenously (50 mg/kg) and orally (500 mg twice daily for 3 months) had a positive effect on the dopaminergic system in patients, significantly improving the symptoms of PD [39]. In animal studies, quercetin, a natural antioxidant abundant in fruits and vegetables, has also been reported to inhibit both increased lipid hydroperoxide levels and decreased dopamine levels by restoring glutathione levels in rat brains treated with 6-OHDA [40]. These previous studies support our proposition that ergothioneine, which can inhibit excessive ROS production or oxidative stress, may be a way to prevent the development and exacerbation of PD. Furthermore, as oxidative stress is a cause of the onset and exacerbation of various diseases, such as other neurological diseases and diabetes [41,42], it is expected that ergothioneine can be applied as a preventive method for other diseases.

As already described, ergothioneine is effective against animal models of Alzheimer’s disease and stroke by exerting antioxidant and neuroprotective effects [17,18]. Neuroprotective effects of ergothioneine have also been reported in studies using cultured cells. For example, ergothioneine inhibits the anticancer drug cisplatin-dependent neuronal cell injury in primary cortical neurons and PC12 cells [43]. While compared with NAC, ergothioneine effectively inhibited the cell death caused by Aβ by inhibiting the formation of peroxynitrite [44]. Other studies have analyzed the efficacy of ergothioneine in other neurological diseases, but to the best of our knowledge, no studies have investigated the efficacy of ergothioneine in animal models of PD or in 6-OHDA-dependent neuronal cell death. Indeed, only one report on the association between PD and ergothioneine has been published; that clinical study reported significantly lower blood levels of ergothioneine in patients with PD compared with age-matched controls. This suggests that low blood levels of ergothioneine may be a risk factor for PD [45]. Based on these results, we believe that supplementation with ergothioneine or ergothioneine-containing foods is an important means of preventing the development and progression of PD.

## 5. Conclusions

In our study, we found that ergothioneine, a rare natural amino acid, prevents 6-OHDA-dependent neuronal cell death by exerting an antioxidant effect. We also found that ergothioneine-rich Rice-koji has similar efficacy as ergothioneine. Based on these results, we propose that ergothioneine may be an effective method to prevent the development and progression of PD. In the future, we would like to clarify the efficacy of ergothioneine or ergothioneine-rich Rice-koji in animal models of PD.

## Data Availability

The data that support the findings of our study are available from the corresponding author upon reasonable request.

## Supplementary Materials

The following supporting information can be downloaded at https://www.mdpi.com/article/10.3390/cells13030230/s1.
