# Supplementary material for: Ergothioneine Prevents Neuronal Cell Death Caused by the Neurotoxin 6-Hydroxydopamine
Source: Cells. 2024 Jan 25;13(3):230. doi: 10.3390/cells13030230 (PMC10854700; doi:10.3390/cells13030230)
Supplement: Supplementary file 1 [file cells-13-00230-s001.zip › cells-2770276-supplementary.pdf]

Name	Forward	Reverse
Gapdh	AACTTTGGCATTGTGGAAGG	ACACATTGGGGGTAGGAACA
Chop	CCACCACACCTGAAAGCAGAA	AGGTGAAAGGCAGGGACTCA
Gadd34	TCCCTCATGGGGAGACTGAA	AGCTGTGCGTTCCATTTCCT
Atf4	GGGTTCTGTCTTCCACTCCA	AAGCAGCAGAGTCAGGCTTTC
Bip	TTCAGCCAATTATCAGCAAACTCT	TTTTCTGATGTATCCTCTTCACCAGT
Ire1a	AGGGGGAGGTCCTGAGAAAG	CTTGGCCTCTGTCTCCTTGG
Pdi	GGATTGCACTGCCAACACAA	AGCTGGTCCTGCTTGTTTCT
Edem	CTACCTGCGAAGAGGCCG	GTTCATGAGCTGCCCACTGA
Grp94	AAGAATGAAGGAAAAACAGGACAAAA	CAAATGGAGAAGATTCCGCC

Supplementary Figure S1. Primer sequences used in this study.

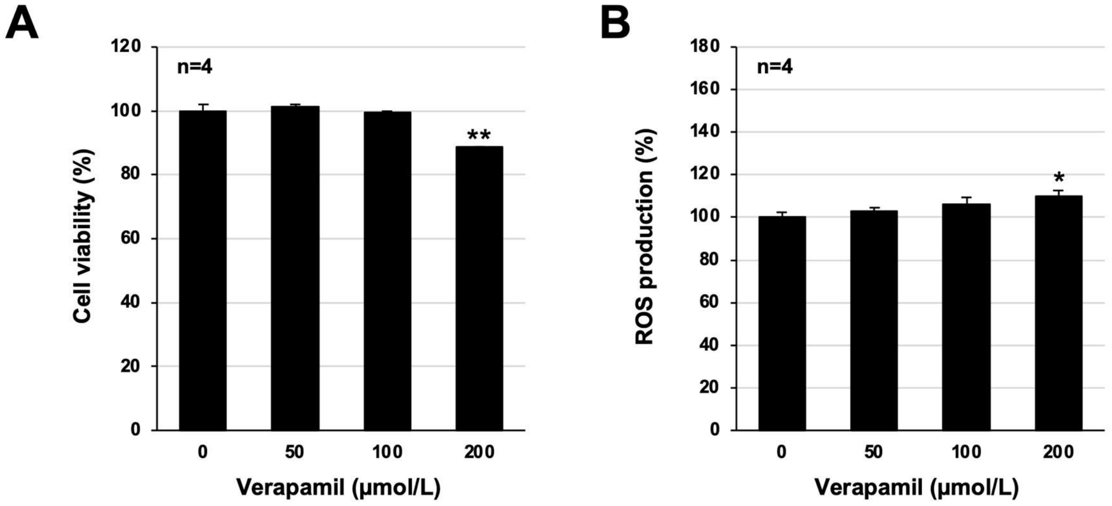

Supplementary Figure S2. Effect of Verapamil Alone Treatment on GT1-7 Cells.
GT1-7 cells were treated with verapamil (0-200 µmol/L) for 60 min. After replacement with fresh medium, cells were incubated for 24 h. Cell viability was measured using CellTiter-Glo® 2.0 (**A**). GT1-7 cells were pretreated with a reactive oxygen species (ROS) indicator, 2′,7′-dichlorodihydrofluorescein diacetate (H_2DCFDA) (10 µmol/L) for 60 min. Then, GT1-7 cells were treated with verapamil (0-200 µmol/L) for 60 min. ROS levels were measured using a fluorescence microplate reader (**B**). Values represent mean ± S.E.M. ($n = 4$). * $p < 0.05$, vs. Control; ** $p < 0.01$, vs. Control.
